# Grants Recommended by the Convalescent Homes Committee

**Published:** 1919-12-20

**Authors:** 


### Grants Recommended by the Convalescent Homes Committee.

*The Convalescent Homes Committee desire to draw attention to the fact that it must not be assumed that the reduction or absence of a grant implies dissatisfaction. (See paragraph 3 of Report.)*

### A.—CONSUMPTION SANATORIA.

Children's Sanatorium, Holt, Norfolk, £880, of which £780 in consideration of the reservation of ten children's beds during 1920 for the use of certain London hospitals (see paragraphs 5 and 6 of Report). Daneswood Sanatorium (Jewish), Woburn Sands, £460, in consideration of the reservation of four beds during 1920 for the use of certain London hospitals (see paragraphs 5 and 6 of Report). Devon and Cornwall Sanatorium, Didworthy, South Brent, £1,250, of which £1,150 in consideration of the reservation of ten beds during 1920 for the use of certain London hospitals (see paragraphs 5 and 6 of Report). Eversfield Chest Hospital, St. Leonards, £50. Fairlight Sanatorium, Hastings, £100. Kelling Sanatorium, Holt, Norfolk, £740, of which £690 in consideration of the reservation of six beds during 1920 for the use of certain London hospitals (see paragraphs 5 and 6 of Report). Mount Vernon Hospital, Northwood, £150. National Association for the Establishment and Maintenance of Sanatoria for Workers, Benenden, Kent, £3,610, of which £750 in reduction of mortgage debt, and £2,760 in consideration of the reservation of twenty-four beds during 1920 for the use of certain London hospitals (see paragraphs 5 and 6 of Report). Northamptonshire Sanatorium, Creaton, Northants, £1,530, of which £1,380 in consideration of the reservation of twelve beds during 1920 for the use of certain London hospitals (see paragraphs 5 and 6 of Report).

**Total for Consumption Sanatoria, £8,770.**

### B.—CONVALESCENT HOMES.

(See note on page 1.)

*The names of Convalescent Homes receiving no grant are not published.*

Beau Site Convalescent Home, Hastings, £75. Brentwood Convalescent Home for London Children, Brentwood, £50. Brooklands Home (Invalid Children's Aid Association), Worthing, £50. Bushey and Bushey Heath Children's Convalescent Home, Bushey, £50. Chelsea Hospital for Women, St. Leonards, £75, with a view to encouraging the admission without payment of patients direct from the hospital. Children's Convalescent Home, Beaconsfield, £25. Children's Cottage Hospital, Cold Ash, Newbury, £25, in consideration of convalescent cases. Children's Home Hospital, Barnet, £25, in consideration of convalescent cases. Convalescent Home for Poor Children, St. Leonards, £150. Convalescent Police Seaside Home, Hove, £75. E. G. Anderson Hospital (Home of Recovery), Barnet, £200. East London Hospital for Children, Bognor, £150, of which £100 to reinstatement after use by military for non-hospital purposes. Florence Emma Home (Invalid Children's Aid Association), Dover, £50. Friendly Societies' Convalescent Homes, Dover and Herne Bay, £75. Great Northern Central Hospital, Clacton, £100. Great Northern Central Hospital, East Finchley, £100. Highgate Convalescent Home for Children, Highgate, £50. Home for Convalescent Children, Southsea, £50. Jewish Convalescent Home (Children), Brighton, £75. King's College Hospital, Hemel Hempstead, £150. The grant is made on condition that patients are admitted free direct from the hospital. Metropolitan Convalescent Institution, Bexhill, £75. Metropolitan Convalescent Institution (Children), Broadstairs, £75. Metropolitan Convalescent Institution, Walton, £150. Middlesex Hospital, Clacton-on-Sea, £50. Miller Hospital Convalescent Home, Bexhill, £100. National Hospital for the Paralysed and Epileptic, East Finchley, £150, with a view to facilitating the admission of patients without payment. Paddington Green Children's Hospital, Slough, £125. Poplar Hospital, Walton-on-Naze, £50. Queen Charlotte's Lying-in Hospital, Kilburn, £75. Queen's Hospital for Children, Bexhill, £100. Royal Waterloo Hospital for Children and Women, Whippingham, £75. St. Andrew's Convalescent Home, Folkestone, £100. St. Andrew's Convalescent Hospital, Clewer, £25. St. Luke's Home (Children), Woodley, Reading, £25, in consideration of convalescent cases. St. Mary's Convalescent Home, Birchington, £50. St. Mary's Home for Children, Broadstairs, £75, in consideration of convalescent cases. St. Michael's, Westgate, £25. Samaritan Free Hospital, Amersham, £55. Sunshine Home, Hurstpierpoint, £25. Surgical Home for Boys, Banstead, £25, in consideration of convalescent cases. Tilford Convalescent Home for Children, Farnham, £50. Victoria Home for Invalid Children, Margate, £25, in consideration of convalescent cases. Victoria Hospital for Children, Broadstairs, £125.

**Total to Convalescent Homes, £3,230.**

| | | |
|---|---|---|
| **Grants to Consumption Sanatoria** | ... | **£8,770** |
| **Grants to Convalescent Homes ...** | ... | **£3,230** |
| **Total Grants for the Year 1919** | ... | **£12,000** |

